# Relationships between Self-Efficacy and Teachers’ Well-Being in Middle School English Teachers: The Mediating Role of Teaching Satisfaction and Resilience

**DOI:** 10.3390/bs14080629

**Published:** 2024-07-23

**Authors:** Xiaochen Wang, Yang Gao, Qikai Wang, Panpan Zhang

**Affiliations:** School of Foreign Studies, Xi’an Jiaotong University, Xi’an 710049, China; wangxiaochen666666@outlook.com (X.W.); tsikay798@163.com (Q.W.); panpanpenny_zhang@163.com (P.Z.)

**Keywords:** self-efficacy, well-being, teaching satisfaction, resilience, middle school English teachers

## Abstract

Teaching satisfaction and resilience play important roles in the education field, but most research focuses on higher education, with few scholars studying their impact on language teaching within the context of middle school education. In this sense, this study employs a mixed-methods research design, selecting 375 Chinese middle school English teachers to investigate the roles of teaching satisfaction and resilience in the relationship between self-efficacy and teachers’ well-being. A structural equation modeling approach and NVivo were utilized to analyze quantitative data and qualitative data, respectively. Quantitative results reveal that both teaching satisfaction and resilience mediate the relationship between self-efficacy and teachers’ well-being. Qualitative interviews reveal that teaching satisfaction primarily enhances job commitment, reduces job stress, improves student relationships, and increases professional growth. Meanwhile, resilience plays a crucial role in stress management, positive adaptation, and emotional regulation. This research offers insightful implications for improving teachers’ well-being and contributes significantly to the broader discourse on foreign language teacher education.

## 1. Introduction

The well-being of English teachers is a critical area of study due to the significant impact on their professional performance and the overall quality of education [1]. Teachers’ well-being influences their motivation, teaching effectiveness, and job satisfaction, which in turn affects student outcomes and school environments [2]. In China, the pressure on English teachers is particularly high due to the emphasis on English proficiency for academic and career success [3]. Additionally, they face challenges such as large class sizes, extensive administrative duties, and high expectations from parents and society [4]. Understanding the factors that contribute to their well-being, such as self-efficacy, teaching satisfaction, and resilience, can inform policies and practices aimed at improving teacher support and development. This research is thus vital for fostering a sustainable and effective educational system that promotes both teacher and student success, ultimately benefiting the broader educational landscape in China.

In the past decades, scholars have studied English teachers’ well-being from several angles: workload and stress [5], job satisfaction [6], professional development [7], and cultural and contextual factors [8]. These angles provide a comprehensive view of factors influencing English teachers’ well-being. However, most research has two main limitations: it primarily focuses on teachers in higher education, with insufficient attention given to those in middle school education. Additionally, existing studies tend to rely solely on either quantitative or qualitative methods, with few employing a mixed-methods approach. This gap highlights the need for comprehensive research that includes middle school teachers and integrates both quantitative and qualitative data to provide a more nuanced understanding of teachers’ well-being.

To fill this research gap, our study adopts a mixed-methods approach to examine the mediating role of teaching satisfaction and resilience in the relationship between self-efficacy and the well-being of middle school English teachers. This study introduces two notable innovations: Firstly, it adopts a mixed-methods approach, integrating both quantitative and qualitative data to offer a more exhaustive and nuanced analysis. Secondly, it focuses explicitly on Chinese middle school English teachers, thereby addressing a gap in existing research that has previously overlooked this demographic. By integrating these methods and focusing on this particular group, our study aims to offer a nuanced understanding of the factors influencing their well-being and contribute valuable insights for educational policies and practices. The research questions are as follows:

Do teaching satisfaction and resilience mediate the relationships between self-efficacy and teachers’ well-being in middle school English teachers? If so, what mediating roles do they play?

## 2. Literature Review

### 2.1. Teachers’ Self-Efficacy

The concept of self-efficacy originates from the social cognitive theory of behavioral change [9]. It refers to a teacher’s belief in their ability to successfully manage tasks, obligations, and challenges related to their professional role [10]. Bandura (1997) divided self-efficacy into four domains: mastery experience, vicarious experience, social persuasion, and psychological and affective or somatic states [11]. Positive mastery or vicarious experiences, along with supportive social or work environments, can enhance self-efficacy, while failures may diminish it [12]. However, the perception of an experience as a success or failure is influenced by various factors, including personal, social, and situational ones [12]. In the context of education, Tschannen-Moran et al. (1998) explain that teachers’ self-efficacy refers to their belief in their ability to effectively plan and execute teaching tasks within specific contexts [13].

Recent studies have examined the relationships between teachers’ self-efficacy and various outcomes in the educational context. Research indicates that higher levels of self-efficacy are associated with better student outcomes, greater job satisfaction, and lower levels of burnout among teachers [14,15]. Studies also suggest that self-efficacy can influence a teacher’s resilience, enabling them to cope more effectively with the stresses and demands of the profession [16,17]. For example, teachers with high self-efficacy are more inclined to perceive challenges as avenues for growth and development rather than as insurmountable barriers [18]. Finally, self-efficacy has been linked to overall well-being, encompassing emotional, psychological, and social aspects of teachers’ lives [19]. Based on these findings, the following hypotheses are proposed:

**H1:**  *Self-efficacy significantly predicts teaching satisfaction.*

**H2:**  *Self-efficacy significantly predicts teacher resilience.*

**H3:**  *Self-efficacy significantly predicts teacher well-being.*

### 2.2. Teaching Satisfaction

Teaching satisfaction can be understood as a specific facet of teachers’ job satisfaction, focusing on their attitudinal and affective responses to teaching [20]. Its significance in the field of education has been extensively documented in various studies [21,22]. Over the past few decades, research on teaching satisfaction has evolved dramatically. Early studies primarily examined the impact of extrinsic factors, such as salary and working conditions, on job satisfaction [23]. However, more recent research has broadened this scope to include intrinsic factors, such as the sense of achievement, professional growth, and the autonomy teachers experience in their classrooms [24]. Recent studies have also investigated the factors influencing teaching satisfaction and its impact on teachers’ overall well-being [1,6]. Specifically, teachers who report high levels of teaching satisfaction experience lower stress levels, higher resilience, and greater overall well-being [25,26]. Given this robust evidence, we hypothesize that teaching satisfaction plays a pivotal role in predicting teacher well-being. Therefore, the following hypothesis is proposed:

**H4:**  *Teaching satisfaction significantly predicts teacher well-being.*

### 2.3. Teacher Resilience

Initially, the term “resilience” described the capacity of individuals to adapt and thrive despite adversity (Masten et al., 1990) [27]. In the emerging field of research on teacher resilience, the concept has been defined in various ways [28,29]. Wagnild and Young (1993) defined resilience as a personality characteristic that moderates the negative effects of stress and promotes adaptation [30]. Bobek (2002) described it as a developmental process occurring over time, including the ability to adjust to different situations and develop competence in difficult conditions [28]. Additionally, teacher resilience can be defined as specific strategies that individuals employ when encountering challenging situations [31]. Over the past two decades, resilience has received considerable attention in teacher education [32,33,34,35]. Recent research highlights its crucial role in sustaining teachers’ well-being. For instance, studies have shown that resilient teachers are better equipped to manage stress, maintain a positive outlook, and engage in reflective practices that promote continuous improvement [18,36]. These findings suggest that resilience not only helps teachers cope with immediate challenges but also fosters a sense of professional fulfillment and well-being over the long term. Thus, the following hypothesis is proposed:

**H5:**  *Resilience significantly predicts well-being.*

### 2.4. Subjective Well-Being Theory as the Framework

Well-being is a complex construct encompassing both affective and psychological functioning, viewed from two distinct perspectives: the hedonic perspective, which focuses on subjective experiences of happiness and life satisfaction, and the eudaimonic perspective, which emphasizes psychological functioning and self-realization [37]. Tennant et al. (2007) conceptualized well-being as an interdisciplinary combination of both the feeling and functioning aspects of mental well-being [37]. The feeling aspect reflects the idea that well-being is a state that varies according to the situation, while the functioning aspect is linked to the notion that well-being is a relatively stable psychological trait. This trait can diminish due to external events or improve through therapeutic interventions.

**Figure 1:**
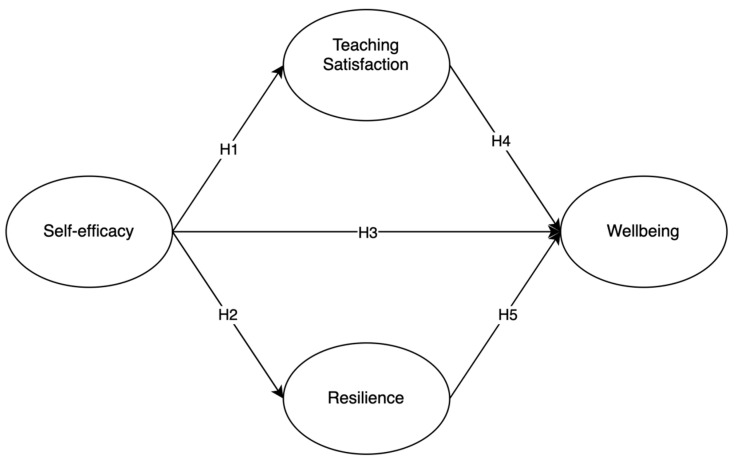
The hypothesized structural model.

Subjective well-being theory is a central concept in positive psychology and consists of three distinct but inter-related components: (1) frequent positive affect; (2) infrequent negative affect; (3) cognitive evaluations of one’s life satisfaction [38]. Tov and Diener (2013) pointed out that there are probably some universal causes for subjective well-being but that differences exist between cultures as well as in its causes and correlates, including socioeconomic status, age, autonomy, self-concepts, and personality traits [39]. In the context of middle school English teachers, subjective well-being is closely linked to teaching satisfaction, self-efficacy, and resilience. Teaching satisfaction reflects the frequent positive affect component, as teachers who enjoy and find fulfillment in their roles experience frequent positive emotions [40]. Self-efficacy, or the belief in one’s ability to execute tasks effectively, influences both positive and negative affects by enhancing confidence and reducing stress [2]. Resilience, the ability to recover from setbacks, corresponds to cognitive evaluations of life satisfaction, enabling teachers to maintain a positive outlook and long-term job satisfaction [41]. These dimensions collectively support the well-being of teachers. Specifically, teaching satisfaction and resilience each play mediating roles. Teaching satisfaction acts as a mediator because it transforms the confidence and effectiveness derived from self-efficacy into enhanced job satisfaction, which in turn boosts overall well-being [6,14,15]. Resilience, on the other hand, mediates the relationship by enabling teachers to use their self-efficacy to effectively manage stress and recover from setbacks, thereby maintaining a positive outlook and emotional stability that supports sustained well-being (see Figure 1) [16,18,36].

## 3. Methodology

### 3.1. Participants

This study employed a mixed-methods design involving both quantitative and qualitative approaches. The quantitative phase involved 375 Chinese middle school English teachers, recruited online. Participants were selected based on two primary criteria: actively teaching English in Chinese middle schools and voluntarily agreeing to participate after understanding the research design, purpose, and procedures. Of the participants, 132 (35.2%) were male and 243 (64.8%) were female. Regarding educational qualifications, 227 (60.5%) held a Bachelor’s degree, 141 (37.6%) had a Master’s degree, and 7 (1.9%) possessed a PhD. In terms of teaching experience, 177 (47.2%) had 5 years or less, 123 (32.8%) had between 5 and 10 years, and 75 (20%) had over 10 years. This diverse sample provided a comprehensive perspective on the study’s research questions. Following the ethical guidelines outlined by the International TESOL Association, the confidentiality of demographic information was guaranteed to participants.

For the qualitative phase, a purposive sampling method was employed to select 15 teachers from the initial pool of 375 participants, ensuring a diverse representation in terms of gender, educational background, and teaching experience. Specifically, the selection criteria included gender balance to reflect the overall composition of the participant pool, varied educational qualifications to include teachers with Bachelor’s, Master’s, and PhD degrees, and a range of teaching experiences from novice (less than 5 years) to veteran (more than 10 years). These criteria were designed to capture a wide array of perspectives and experiences, thereby enriching the qualitative findings. The selected teachers voluntarily agreed to participate in in-depth interviews, providing detailed insights into their teaching satisfaction and resilience. This approach ensured that the qualitative data were robust and reflective of the diverse experiences of Chinese middle school English teachers.

### 3.2. Instruments

#### 3.2.1. The Self-Efficacy Scale

In the current study, we employed a modified version of the self-efficacy questionnaire originally developed by Klassen et al. (2009) to specifically assess the self-efficacy of middle school English teachers [42]. This adaptation involved tailoring the questionnaire to the unique context of English language teaching at the middle level. The revised instrument consists of 12 items distributed across three dimensions: “Self-efficacy for instructional strategies”, “Self-efficacy for student engagement”, and “Self-efficacy for classroom management”. Each item is evaluated using a five-point Likert scale, with response options ranging from “strongly disagree” (1) to “strongly agree” (5). This scale allows for the quantification of teachers’ perceived efficacy in managing instructional challenges, engaging students, and maintaining classroom discipline, thereby providing a comprehensive measure of their professional confidence.

#### 3.2.2. The Resilience Scale

Resilience among middle school English teachers was measured using a modified version of the resilience questionnaire originally devised by Campbell-Sills and Stein [43]. This adaptation was tailored to meet the specific requirements of the study, with a focus on the resilience characteristics pertinent to middle school English educators. The revised questionnaire comprises 10 items, each rated on a five-point Likert scale. This scale spans from “strongly disagree” (1) to “strongly agree” (5), enabling the assessment of the degree to which participants agree with statements reflecting resilience. This approach facilitates the evaluation of the teachers’ capacity to endure and adapt to the professional challenges unique to their educational settings.

#### 3.2.3. The Teaching Satisfaction Scale

In the present study, the construct of teaching satisfaction among middle school English teachers was assessed using a customized version of the questionnaire originally created by Ho and Au [20]. Initially designed for both primary and middle school teachers, the questionnaire was specifically adapted to focus on middle school English teachers to align with the study’s particular needs. The modified questionnaire contains five items, each measured on a five-point Likert scale. This scale ranges from “strongly disagree” (1) to “strongly agree” (5), allowing for the collection of detailed responses regarding teachers’ levels of satisfaction with their teaching experiences. This tailored approach enables a focused analysis of the factors contributing to teaching satisfaction within this specific context.

#### 3.2.4. The Well-Being Scale

In this research, the well-being of middle school English teachers was evaluated using an adapted version of the well-being questionnaire initially developed by Mankin et al. (2018) [44], which was originally aimed at teachers from six states in the United States. To suit the specific focus of this study, the questionnaire was modified to assess the well-being of middle school English teachers exclusively. This tailored version includes eight items, each assessed on a five-point Likert scale. The response options for this scale range from “strongly disagree” (1) to “strongly agree” (5). This modification allows for precise measurement of the well-being levels of these teachers, capturing their unique experiences and challenges in the educational sector.

#### 3.2.5. Semi-Structured Interview

The semi-structured interviews aimed to explore the nuances of how teaching satisfaction and resilience mediate the relationships between self-efficacy and teachers’ well-being in middle school English teachers. Specific questions included the following: in terms of teaching satisfaction, the questions about how satisfaction with teaching influences overall well-being; the impact of feeling satisfied or dissatisfied with teaching on self-efficacy in the classroom; the contribution of positive teaching experiences to effectiveness and happiness; examples of how teaching satisfaction has enhanced or diminished well-being; and the connection between teaching satisfaction and work–life balance. Regarding resilience, the questions addressed coping with challenges and setbacks in teaching and its effect on well-being; instances where resilience helped maintain or improve self-efficacy; the influence of bouncing back from difficulties on job satisfaction and personal happiness; the impact of building resilience on well-being and teaching effectiveness; and the role of resilience in maintaining motivation and enthusiasm for teaching over time.

### 3.3. Data Collection

Data collection was conducted in two sequential phases to ensure a robust collection of both quantitative and qualitative data. Initially, an online survey was disseminated through Wenjuanxin, which ensured a broad reach among potential participants. Following this, a purposive sample of 15 respondents was selected for semi-structured interviews. This qualitative phase aimed to delve deeper into individual experiences, offering rich, detailed accounts that complemented the numerical data. The interviews were conducted primarily in Chinese, with each session lasting approximately 30 min.

### 3.4. Data Analysis

The data analysis for this study was executed through both quantitative and qualitative lenses, utilizing SPSS 26.0 and AMOS 26.0 for the former and NVivo 12.0 for the latter. Quantitative data were processed in a multi-step approach following structural equation modeling guidelines [45,46,47]. Initially, data cleaning was performed to remove invalid responses and detect outliers using Mahalanobis distance, and skewness and kurtosis values were examined to ensure normal distribution. Reliability and validity assessments followed, with Cronbach’s alpha coefficients calculated to verify internal consistency, and confirmatory factor analysis conducted to validate the measurement model. Descriptive statistics provided insights into the variability and central tendencies of the data points. SPSS analysis facilitated testing the interrelationships between the constructs of self-efficacy, teaching satisfaction, resilience, and teacher well-being. Finally, a SEM analysis was conducted to ascertain any mediating effects on the hypothesized pathways. The qualitative data analysis was primarily conducted using NVivo 12.0, involving three main steps: (1) reviewing and translating participants’ responses to ensure accurate comprehension; (2) coding by units of words, relevant phrases, and sentences related to each of the factors at the nodes created for emerging research themes; (3) summarizing the themes based on the frequency of the nodes to address the research questions.

## 4. Results

### 4.1. Descriptive Analysis

Table 1 demonstrates the means (M) and Standard Deviations (SD) for the items measured by the five-point Likert Scale. To be exact, the self-efficacy scores indicate moderate confidence in their teaching abilities, with mean scores between 3.65 and 4.20. Teaching satisfaction shows varied levels of contentment, with means ranging from 3.55 to 4.07. The resilience scores suggest moderate resilience levels, with means between 3.39 and 4.01. The well-being scores indicate a moderate level of overall well-being, with means from 3.63 to 3.94.

### 4.2. Reliability and Validity Checks and the Measurement Model

This section sequentially presents the results related to reliability, multivariate normality, convergent validity, discriminant validity, and the measurement model. To evaluate internal consistency reliability, Cronbach’s α values were calculated for the nine variables (see Table 2). The results revealed Cronbach’s α values as follows: 0.93 for self-efficacy, 0.86 for teaching satisfaction, 0.92 for resilience, and 0.92 for well-being. These values were all significantly higher than the widely accepted benchmark of 0.7, as recommended by Kline (2023) [46], which indicates a high level of reliability for the scales employed in this research.

Secondly, an evaluation of the multivariate normality and the adequacy of sampling was conducted using two well-established statistical tests: the Kaiser–Meyer–Olkin (KMO) measure and Bartlett’s test of sphericity. The results indicated a significant value for Bartlett’s test of sphericity (*p* < 0.001), alongside a KMO value of 0.78. These findings suggest that the data were suitable for conducting factor analysis, as per the guidelines provided by Tabachnick and Fidell (2019) [48]. In addition to these assessments, the univariate skewness and kurtosis values for all items on the scales were calculated to further verify the normality of the dataset. As presented in Table 2, the absolute values of skewness and kurtosis for all items were below 2, respectively. According to the criteria outlined by Collier (2020) [45], these values confirm that the data exhibit normal distribution characteristics, thereby supporting the appropriateness of the data for subsequent statistical analyses.

Furthermore, a confirmatory factor analysis (CFA) was conducted to examine the validity of the measurement model. In line with the procedures recommended by Collier (2020), both composite reliability (CR) and average variance extracted (AVE) scores were calculated for each factor to assess convergent validity. The results, as presented in Table 2, showed that the CR values for all factors exceeded the recommended threshold of 0.7, while the AVE values for each factor were above the 0.5 benchmark. These findings confirm that the factors demonstrate strong convergent validity, as suggested by Kline (2023) [46]. To further evaluate discriminant validity, the square roots of the AVE values were compared with the inter-factor correlation coefficients for the nine factors. The analysis revealed that the square root values of AVE were consistently higher than the squared correlations between constructs, which provides additional evidence for discriminant validity. This rigorous approach to assessing both convergent and discriminant validity ensures that the measurement model is robust and reliable, supporting the validity of the constructs used in this study.

To further examine construct validity, a comprehensive measurement model was developed using AMOS 26.0 software. The model fit was meticulously assessed by computing six key goodness-of-fit indices: the comparative fit index (CFI), normed fit index (NFI), incremental fit index (IFI), root mean square error of approximation (RMSEA), Tucker–Lewis index (TLI), and standardized root mean squared residual (SRMR). As shown in Table 3, the results indicated that our model demonstrated an excellent fit with the data. Specifically, all computed indices met or exceeded the recommended benchmark values, as established by Collier (2020), Kline (2023), and Wheaton et al. (1977) [45,46,49].

### 4.3. The Structural Model and Hypotheses Testing

**Figure 2:**
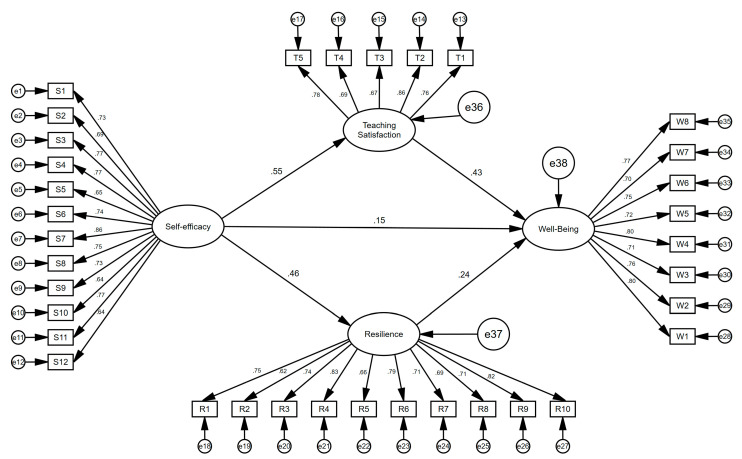
The final model.

Building on the established measurement model, we tested the full structural model, which demonstrated a good fit based on the goodness-of-fit statistics in Figure 2. Summarizing the major results of the path analysis, Table 4 details the acceptance of all five hypotheses.

Table 5 presents the mediation analysis results of the relationships between self-efficacy (S), teaching satisfaction (T), resilience (R), and well-being (W). The analysis reveals two significant mediation paths: S→T→W and S→R→W. For the path S→T→W, the indirect effect is 0.299 with a 95% confidence interval ranging from 0.186 to 0.413 (*p* = 0.016), indicating that teaching satisfaction significantly mediates the relationship between self-efficacy and well-being. Similarly, for the path S→R→W, the indirect effect is 0.137 with a 95% confidence interval from 0.083 to 0.241 (*p* = 0.004), indicating that resilience also significantly mediates the relationship between self-efficacy and well-being.

### 4.4. The Role of Teaching Satisfaction and Resilience

#### 4.4.1. The Role of Teaching Satisfaction

Firstly, teaching satisfaction plays an important role in enhancing job commitment. Teaching satisfaction reinforces a teacher’s commitment to their job. When teachers feel effective (self-efficacy), their satisfaction with teaching increases, making them more committed to their profession [14]. This commitment enhances their overall sense of well-being by providing a sense of purpose and fulfillment [15]. Specific interview examples are as follows:

P1: I find that when I’m satisfied with my teaching, I feel a stronger commitment to my job. It gives me a sense of purpose that motivates me to do my best every day.

P4: Feeling effective in my teaching role makes me more dedicated to my profession. It’s like a positive cycle—satisfaction leads to commitment, which in turn boosts my overall happiness.

P12: Teaching satisfaction definitely reinforces my job commitment. Knowing that I’m making a difference keeps me dedicated to my profession, which improves my overall well-being.

Secondly, teaching satisfaction holds a key role in reducing job stress. Higher teaching satisfaction can reduce job-related stress. Teachers with high self-efficacy who are satisfied with their teaching roles are less likely to feel overwhelmed by job demands, leading to lower stress levels and improved well-being. Examples from the interviews are provided below:

P2: High teaching satisfaction helps me manage stress better. I’m less overwhelmed and more able to enjoy my work, leading to better well-being.

P7: I’ve noticed that when my satisfaction with teaching is high, my job stress is significantly lower. It helps me stay calm and focused, which is crucial for my mental health.

P14: My job-related stress decreases when I’m satisfied with my teaching. It helps me cope with the demands more effectively, improving my overall well-being.

Thirdly, teaching satisfaction is fundamental to improving student relationships. Teaching satisfaction positively affects the relationships between teachers and students. Self-efficacious teachers who are satisfied with their teaching are more likely to engage positively with students, fostering a supportive classroom environment that enhances their well-being. The following are specific examples from the interviews:

P3: When I’m satisfied with my teaching, I engage more positively with my students. This improved relationship boosts my happiness and job satisfaction.

P9: Teaching satisfaction enhances my interactions with students. When I feel effective, I’m more patient and supportive, which benefits both them and me.

P15: Being satisfied with my teaching role positively impacts my student relationships. It makes me more approachable and effective, enhancing my overall job satisfaction.

Lastly, teaching satisfaction is crucial in increasing professional growth. Satisfied teachers are more likely to seek out professional development opportunities. When teachers believe in their abilities (self-efficacy) and are satisfied with their teaching, they are motivated to grow professionally, which contributes to their sense of accomplishment and well-being. Here are detailed examples from the interviews:

P5: Teaching satisfaction motivates me to seek out professional development opportunities. It’s important for my growth and enhances my sense of accomplishment.

P8: When I’m satisfied with my teaching, I’m more inclined to pursue further training. This professional growth contributes significantly to my well-being.

P13: High teaching satisfaction leads me to seek out new learning opportunities. This professional growth is rewarding and enhances my job satisfaction.

#### 4.4.2. The Role of Resilience

Resilience plays an important role in stress management. Resilience helps teachers manage stress effectively, which is crucial for maintaining well-being. When teachers have high self-efficacy, they believe in their ability to handle classroom challenges [36]. This belief fosters resilience, enabling them to cope with stress and reduce burnout, leading to better overall well-being [18]. Specific interview examples are as follows:

P1: Believing in my ability to manage my classroom effectively makes it easier to cope with stressful situations. This resilience keeps me from burning out.

P4: I find that my self-efficacy helps me to stay focused and not get overwhelmed by the workload. This resilience is essential for managing stress and maintaining my well-being.

P7: Knowing I can handle difficult classroom scenarios reduces my stress levels. This belief in my capabilities fosters resilience and helps me maintain a positive outlook.

Secondly, resilience holds significant importance in positive adaptation. Resilient teachers can adapt positively to adverse situations. High self-efficacy boosts their confidence in overcoming obstacles. This positive adaptation reduces feelings of helplessness and enhances their sense of accomplishment and satisfaction, thereby improving their well-being. Detailed interview examples include the following:

P2: I adapt quickly to changes and challenges in the classroom because I trust my skills. This positive adaptation makes me feel more accomplished and satisfied with my job.

P8: I feel capable of handling any situation that comes my way. This ability to adapt positively enhances my sense of achievement and happiness.

P12: I don’t feel helpless when things go wrong. My resilience, supported by my confidence in my teaching abilities, helps me adapt and stay satisfied with my work.

Resilience plays a vital role in emotional regulation. Resilience aids in emotional regulation, allowing teachers to maintain emotional stability. Self-efficacious teachers can leverage resilience to control their emotional responses to stressful situations. Effective emotional regulation minimizes negative emotions and enhances positive feelings, thereby promoting well-being. Here are concrete examples from the interviews:

P4: Resilience helps me keep my emotions in check, even on the most stressful days. My self-efficacy ensures that I stay emotionally balanced.

P11: I can manage my emotions better because I believe in my teaching abilities. This resilience is key to maintaining emotional stability and well-being.

P15: When faced with difficult students or situations, my confidence allows me to stay calm and regulate my emotions effectively. This emotional stability is crucial for my well-being.

## 5. Discussion

### 5.1. Teaching Satisfaction and Resilience: Keys to Teachers’ Well-Being

Our study reveals that teaching satisfaction significantly mediates the relationship between self-efficacy and well-being among middle school English teachers. This finding aligns with Matteucci et al. (2017) [15], who noted that teachers with high self-efficacy tend to experience greater job satisfaction, which in turn enhances their overall well-being. The mediating role of teaching satisfaction suggests that when teachers feel competent and effective in their roles, they are more likely to derive satisfaction from their work [14]. This satisfaction then contributes positively to their well-being, as it reinforces their commitment and positive attitudes towards teaching [6]. Compared to previous studies that primarily focused on higher education instructors [1], our research extends these findings to the middle education context, highlighting the universal importance of teaching satisfaction across different educational levels. The significance of this mediating role underscores the need for policies and practices that boost teacher efficacy and satisfaction. By providing professional development opportunities, supportive leadership, and a positive work environment, schools can enhance teachers’ job satisfaction, ultimately leading to better well-being and more effective teaching.

In addition, our study finds that resilience plays a crucial mediating role in the relationship between self-efficacy and well-being. This is consistent with the findings of Wang et al. (2022) as well as Wang and Pan (2023) [17,18], who emphasized that resilient teachers are better equipped to handle the stresses and challenges of the teaching profession. Resilience enables teachers to maintain a positive outlook and persist in the face of adversity, which in turn supports their well-being [19]. This mediating effect suggests that self-efficacy not only boosts teachers’ confidence in their abilities but also fosters resilience, allowing them to navigate challenges more effectively [16]. Compared to earlier research focusing on higher education, our study highlights the critical role of resilience in middle education, where teachers often face unique stressors such as adolescent behavior management and testing pressures. The implications of this finding are significant for educational policy and practice. By incorporating resilience-building strategies into teacher training programs and providing ongoing support, schools can help teachers develop the resilience needed to sustain their well-being and effectiveness. This approach can lead to more stable and satisfied teaching staff, ultimately benefiting the broader educational community.

### 5.2. Resilience and Self-Efficacy: Pillars of Teacher Well-Being

Our study highlights that teaching satisfaction significantly mediates the relationship between self-efficacy and well-being among middle school English teachers. Firstly, teaching satisfaction enhances job commitment, as teachers who feel effective in their roles are more satisfied and thus more committed to their profession. This commitment provides a sense of purpose and fulfillment, which enhances overall well-being. This finding is consistent with Capone et al. (2022) [36], who noted that job satisfaction is crucial for maintaining high levels of teacher well-being. Secondly, higher teaching satisfaction is associated with reduced job-related stress. Teachers with high self-efficacy who are satisfied with their teaching roles feel less overwhelmed by job demands, leading to lower stress levels and improved well-being. This aligns with Klassen and Chiu (2010), who found that job satisfaction is inversely related to teacher stress [50]. Thirdly, teaching satisfaction improves student relationships. Satisfied and self-efficacious teachers are more likely to engage positively with students, creating a supportive classroom environment that enhances their well-being. This supports the findings of Poulou (2020), who emphasized the positive impact of teaching satisfaction on student–teacher relationships [51]. Lastly, teaching satisfaction fosters professional growth. Teachers who are satisfied with their roles and believe in their abilities are more motivated to pursue professional development opportunities, contributing to their sense of accomplishment and well-being. This is in line with the work of Meyer et al. (2023), who highlighted the importance of continuous professional development for teaching satisfaction and effectiveness [52].

In addition, our study underscores the significant mediating role of resilience in the relationship between self-efficacy and well-being among middle school English teachers. Firstly, resilience plays a crucial role in stress management. Teachers with high self-efficacy believe in their ability to handle classroom challenges, which fosters resilience. This resilience enables them to manage stress effectively and reduce burnout, ultimately enhancing their overall well-being. This finding aligns with Wang et al. (2022), who highlighted that resilient teachers are better equipped to handle stress and maintain their well-being [18]. Secondly, resilience promotes positive adaptation to adverse situations. Teachers with high self-efficacy are confident in overcoming obstacles, which enhances their resilience. This positive adaptation helps reduce feelings of helplessness and boosts their sense of accomplishment and satisfaction, thereby improving their well-being. This observation supports the research of Jennings et al. (2013), who found that resilience is essential for teachers to adapt to the dynamic demands of the classroom environment [53]. Thirdly, resilience aids in emotional regulation, allowing teachers to maintain emotional stability. Self-efficacious teachers can leverage resilience to control their emotional responses to stressful situations. Effective emotional regulation minimizes negative emotions and enhances positive feelings, promoting overall well-being. This is consistent with the findings of Hu (2023), who noted that resilient teachers are better at managing their emotions, which is crucial for sustaining their well-being [54].

### 5.3. Theoretical and Practical Implications

Theoretically, this study makes significant contributions to the subjective well-being theory. By focusing on middle school English teachers in China, it extends the application of the theory, highlighting the universal relevance of well-being constructs across different educational levels and cultural settings. Moreover, it identifies the mediating roles of teaching satisfaction and resilience in the relationship between self-efficacy and well-being, providing a nuanced view of the mechanisms at play. Furthermore, by integrating findings from resilience, self-efficacy, and teaching satisfaction, the study offers a practical framework for applying subjective sell-being theory in educational settings. This framework can be used to develop targeted interventions aimed at enhancing teacher well-being and effectiveness. Thus, this study not only contributes to the theoretical foundations of subjective well-being theory but also offers practical insights for improving educational outcomes.

In addition, the study offers several practical implications for enhancing the well-being of middle school English teachers through the dimensions of resilience, self-efficacy, and teaching satisfaction. Firstly, schools should incorporate resilience-building strategies into teacher training programs, providing ongoing support to help teachers manage stress, adapt to challenges, and maintain a positive outlook [55,56,57]. Additionally, enhancing teachers’ self-efficacy through professional development opportunities and supportive leadership can boost their confidence and classroom effectiveness, which in turn leads to higher job satisfaction and well-being [58,59,60]. Moreover, improving the work environment, ensuring adequate resources, and fostering a supportive administrative structure can significantly enhance teaching satisfaction [61,62]. This not only reduces job-related stress but also fosters stronger teacher–student relationships and encourages professional growth. By addressing these dimensions holistically, educational institutions can create a supportive and effective teaching environment that promotes both teacher and student success.

## 6. Conclusions

This study employs a mixed-methods research design, integrating structural equation modeling and interview data to explore the mediating roles of teaching satisfaction and resilience in the relationships between self-efficacy and teachers’ well-being among middle school English teachers. The quantitative findings indicate that both teaching satisfaction and resilience serve as mediators. The qualitative data further elucidate that teaching satisfaction primarily enhances job commitment, reduces job stress, improves student relationships, and increases professional growth. Meanwhile, resilience plays crucial roles in stress management, positive adaptation, and emotional regulation.

This research also has some shortcomings. Firstly, the quantitative data collection relies exclusively on online questionnaires, which may not capture the full spectrum of participants’ experiences and perspectives, thus limiting the depth and breadth of data. Secondly, the study lacks a longitudinal approach to observe and analyze the evolution of teaching satisfaction and resilience among participants. This absence of long-term tracking means potential dynamic changes and trends in these key variables over time are not explored, omitting valuable insights into how teaching satisfaction and resilience might develop or fluctuate in the long term. Future studies should expand their data collection methods beyond online questionnaires to include interviews, observations, and diary entries, offering a richer, more nuanced understanding of middle school English teachers’ teaching satisfaction and resilience. The need for longitudinal research is also highlighted, as tracking middle school English teachers over time can reveal how teaching satisfaction and resilience evolve, providing insights into periods or factors that significantly impact their development.

## Figures and Tables

**Table 1:** Descriptive statistics.

Constructs	Items	M	SD	Skewness	Kurtosis
Self-efficacy	S1	3.97	0.93	−0.64	−0.33
	S2	4.00	0.95	−0.88	0.54
	S3	4.20	0.86	−1.00	0.60
	S4	3.82	0.94	−0.74	0.32
	S5	3.65	1.00	−0.60	−0.07
	S6	3.85	0.91	−0.72	0.16
	S7	3.72	0.96	−0.60	−0.16
	S8	3.79	0.93	−0.68	0.19
	S9	3.93	1.00	−0.84	0.15
	S10	3.93	0.99	−0.87	0.38
	S11	3.81	0.97	−0.81	0.32
	S12	3.75	1.04	−0.68	−0.10
Teaching Satisfaction	T1	3.77	1.05	−0.74	0.17
	T2	4.07	0.91	−0.85	0.22
	T3	3.69	1.06	−0.64	−0.10
	T4	3.55	1.13	−0.51	−0.37
	T5	3.77	1.09	−0.84	0.24
Resilience	R1	3.75	0.94	−0.74	0.44
	R2	3.77	0.97	−0.70	0.19
	R3	3.58	0.88	−0.57	0.26
	R4	3.41	0.92	−0.77	0.17
	R5	3.76	1.01	−0.71	−0.04
	R6	4.01	0.90	−0.84	0.29
	R7	3.56	0.94	−0.83	0.57
	R8	3.39	0.95	−0.51	−0.16
	R9	3.44	0.98	−0.51	−0.12
	R10	3.58	0.90	−0.80	0.60
Well-being	W1	3.74	1.09	−0.61	−0.33
	W2	3.87	1.05	−0.86	0.22
	W3	3.88	1.01	−0.64	−0.20
	W4	3.85	1.04	−0.63	−0.44
	W5	3.94	1.01	−0.90	0.56
	W6	3.79	1.03	−0.77	0.17
	W7	3.63	1.06	−0.54	−0.19
	W8	3.67	1.06	−0.82	0.26

**Table 2:** Reliability and validity.

AVE	CR	α	-	S	T	R	W
**0.53**	**0.93**	**0.93**	S	**0.73**			
0.57	0.87	0.86	T	0.54	**0.76**		
0.54	0.92	0.92	R	0.45	0.50	**0.74**	
0.58	0.92	0.92	W	0.48	0.62	0.50	**0.76**

Note: S = self-efficacy; T = teaching satisfaction; R = resilience; W = well-being; The square root of AVE is demonstrated along the diagonal line in bold.

**Table 3:** Goodness-of-fit indices of the measurement model.

	X^2^/df	CFI	NFI	IFI	RMSEA	TLI	SRMR
Our Model	1.35	0.97	0.91	0.97	0.03	0.97	0.06
RV	<5	>0.90	>0.90	>0.90	<0.10	>0.90	<0.08

Note: RV = Recommended Values.

**Table 4:** Hypotheses test results.

Hypotheses		Estimate	S.E.	β	*p*	t	Results
H1	S 🡪 W	0.191	0.078	0.15	0.015	2.436	accepted
H2	S 🡪 T	0.652	0.072	0.55	***	9.043	accepted
H3	S 🡪 R	0.477	0.061	0.46	***	7.800	accepted
H4	T 🡪 W	0.460	0.067	0.43	***	6.868	accepted
H5	R 🡪 W	0.287	0.064	0.24	***	4.455	accepted

Note: S = self-efficacy; T = teaching satisfaction; R = resilience; W = well-being; *** *p* < 0.001.

**Table 5:** The mediation analysis.

Path	95% Confidence Interval		*p*	Indirect Effect	Results
	Lower	Upper			
S → T → W	0.186	0.413	0.016	0.299	accepted
S → R → W	0.083	0.241	0.004	0.137	accepted

Note: S = self-efficacy; T = teaching satisfaction; R = resilience; W = well-being.

## Data Availability

The data presented in this study are available upon request from the corresponding author.
